# Beyond diversity recruitment: Next steps to ensure that underrepresented emergency medicine residents thrive

**DOI:** 10.1002/aet2.70037

**Published:** 2025-05-08

**Authors:** Rosemarie Diaz, Adam McFarland, Ryan Tsuchida, Tanesha Beckford, Sandra Coker, Jeremy Collado, Arthur Pope, Jeffrey I. Schneider, Alden Landry, Teresa Y. Smith, Jessica Faiz

**Affiliations:** ^1^ Department of Emergency Medicine University of California Los Angeles Los Angeles California USA; ^2^ Case Western Reserve University Cleveland Ohio USA; ^3^ School of Medicine and Public Health University of Wisconsin Madison Wisconsin USA; ^4^ Mass General Brigham Boston Massachusetts USA; ^5^ UT Health Houston Houston Texas USA; ^6^ Mayo Clinic College of Medicine and Science Jacksonville Florida USA; ^7^ University of Pennsylvania Perelman School of Medicine Philadelphia Pennsylvania USA; ^8^ Boston Medical Center Boston Massachusetts USA; ^9^ Beth Israel Deaconess Medical Center in Boston Massachusetts Boston Massachusetts USA; ^10^ Castle Society, Harvard Medical School Boston Massachusetts USA; ^11^ SUNY Downstate Health Sciences University Brooklyn New York USA

## Abstract

A diverse workforce in emergency medicine (EM) aims to improve patient care by addressing racism in health care, increasing representation in medicine, and improving the quality of training for all residents. Many EM residency programs have launched recruitment efforts to attract residents from diverse backgrounds. However, recruitment efforts only represent the first step in building a culturally responsible workforce. Trainees who are underrepresented in medicine must be welcomed into an inclusive training environment that has been thoughtfully constructed before they arrive. This type of supportive environment can be achieved by shifting away from majority‐serving ideals and building an informed infrastructure that functions to help all trainees succeed. We expand upon challenges and areas of opportunity at the individual, departmental, and institutional levels and describe common pitfalls when trying to create inclusive spaces for residents including lack of vision alignment, inadequate financial investment, and performative allyship. We also propose strategies that focus specifically on actionable changes that residency program, departmental, and institutional leadership can implement to mitigate these challenges.

## INTRODUCTION

Studies have shown that creating a diverse workforce can reduce health disparities, address structural racism in health care, and improve the quality of training for all residents.^1^, ^2^, ^3^, ^4^, ^5^, ^6^, ^7^, ^8^, ^9^, ^10^, ^11^, ^12^ Universal recommendations from the Accreditation Council for Graduate Medical Education (ACGME) and emergency medicine (EM) specialty professional organizations, such as the Council of Residency Directors in Emergency Medicine (CORD), Society for Academic Emergency Medicine (SAEM), and American College of Emergency Physicians (ACEP), all advocate for creating a department and training culture that is diverse, equitable, and inclusive with a strong emphasis on diversity, equity, and inclusion (DEI) in residency recruitment.^11^ Many EM residency programs have launched recruitment initiatives to attract a more diverse residency class. Efforts include offering scholarships to visiting clerkship students, holding diversity interview days, utilizing equitable and inclusive interview practices, and establishing subcommittees focused on the diversity of the applicants who are interviewed and match.^12^ As defined by the Association of American Medical Colleges (AAMC), “underrepresented in medicine” refers to racial and ethnic populations who are underrepresented in the medical profession relative to their numbers in the general population.^13^ However, there are other distinct communities who are underrepresented in the medical field, including those who are LGBTQ+, first‐generation immigrants, and of lower socioeconomic status.^14^ Many of the principles underlying the individual, departmental, and institutional challenges that are outlined here can also apply to these other groups in medicine.

While recruitment efforts designed to attract applicants with underrepresented in medicine (UiM) backgrounds to EM demonstrate a concerted effort to commit to DEI principles, these efforts only represent one critical step in creating a strong diverse workforce. Equal attention should be placed on the retention of trainees who were intentionally recruited. According to data from AAMC from 2001 to 2020, the rate of attrition for UiM and women residents from their training programs was higher than those of residents who identified as non‐Hispanic White trainees and men.^15^, ^16^, ^17^ This disparity should signal the necessity to recognize that differences exist during training for UiM residents, and proactive, preventative measures are needed.^18^ Additionally, programs and institutions that launch diversity recruitment programs must also accept their added responsibility to adequately prepare, design, and adapt their training programs to assure that the learning environment is reflective of the same values that were promoted during diversity recruitment.^19^, ^20^ Leaders at the residency, departmental, and institutional levels must assure their training environment is equipped to support the UiM trainees who they drew to the program and deliver the dedication to DEI that was advertised.^21^ Here, we provide a critical framework informed by the literature that gives specific recommendations to assure that UiM trainees thrive at their training programs. These comprehensive, multilevel recommendations aim to address common challenges at the individual, department, and institutional levels in fostering truly inclusive spaces.

## ADDRESSING INDIVIDUAL‐LEVEL CHALLENGES

Residency leaders must understand the unique needs of UiM trainees to appropriately support them.^22^, ^23^ We summarize distinct themes surrounding the unique experiences of UiM physicians as they navigate majority‐serving training environments, which can have a profound impact on their training experience and threaten their ability to thrive.^24^, ^25^

### Recognize and mitigate the impact of homophily

Homophily is the phenomenon that individuals are more likely to associate with those of similar backgrounds and interests.^26^, ^27^ In the training environment, residents from diverse backgrounds may experience feelings of isolation as they struggle to make connections with other residents and faculty whose interests represent that of majority groups.^28^, ^29^, ^30^, ^31^, ^32^ This may result in the exclusion of UiM residents from social events and academic opportunities and impede their ability to establish a support network, develop strong relationships with colleagues, and receive mentorship and career guidance.^33^, ^34^ Residency programs must recognize any proclivities toward homophily and work to promote inclusion for UiM trainees socially and professionally. Program leaders can foster this sense of belonging through mentorship and community‐building activities.

### Know the cumulative harm of microaggressions

UiM residents experience microaggressions from patients and colleagues that challenge their legitimacy as physicians and can negatively affect their wellness.^35^, ^36^ These microaggressions can take the form of being mistaken for a colleague of the same race, being subject to personal questions about their racial background (e.g., “Where are you *really* from?”), and being mistaken for ancillary staff despite introducing themselves as physicians and wearing badges that prominently display their medical degree.^37^, ^38^ Training on recognizing and addressing microaggressions should be an integral component of the residency program educational curriculum, as it has been shown to promote inclusive behaviors, enhance trainees' sense of belonging, and encourage community members to take on the challenging role of being an upstander—one who intervenes or acts in support of trainees.^39^, ^40^, ^41^, ^42^

### Understand the impact of racism

UiM residents also report overt racism during clinical shifts, such as being called derogatory racist terms, “fired” by patients due to their background, or labeled with racially biased traits (e.g. angry, intimidating, unfriendly).^35^ Experiences of racism during residency has been shown to have negative impacts on the physical and mental health of medical trainees.^37^ Pressures to avoid these encounters can lead trainees to code‐switch, that is, change their appearance, speech, or behavior to not appear as “outsiders” or “other.” This external pressure to cover or hide their unique identities and diverse perspectives works against how these features are what may have contributed to the strength of their application during the recruitment process.^41^ This pressure to code switch can lead to internal conflict and dissonance as UiM trainees attempt to reconcile their personal identity with becoming a physician in a majority‐dominated medical culture.^43^

There should be a zero‐tolerance policy for racism and racist experiences should be immediately address by leadership. All physicians and health care providers should feel protected from mistreatment. Clear reporting mechanisms are needed where UiM trainees, or allies who witness racist encounters, can report anonymously without fear of retaliation. Of note, these encounters may come from interactions with patients themselves or colleagues, and no matter the source the act of racism should not be tolerated.^44^

### Recognize the external cues that feed imposter syndrome

Cumulative forces push UiM residents to the periphery of residency culture by creating a sense of “other” and threatening a sense of belonging. This facilitates the development of imposter syndrome in many UiM residents, which results in trainees doubting their qualifications and skills despite being high‐achieving physicians.^45^, ^46^, ^47^, ^48^ While imposter syndrome is traditionally seen as an internal discrepancy within an individual that results in self‐doubt despite professional accomplishments, the term imposter “phenomenon” acknowledges that we must also critically look outward toward the learning environment that majority‐serving institutions create that may trigger these individual feelings.^49^ For example, being labeled a “DEI hire” can perpetuate feelings of inadequacy and doubt, triggering imposter syndrome by undermining an individual's perception of their qualifications, and reinforcing the belief that their achievements are attributed solely to diversity initiatives rather than merit. Experiences of bias and microaggressions during medical on can further accentuate feelings of imposter phenomenon and challenge professional identity formation.^50^, ^51^ Program leadership must build an inclusive environment that instills a sense of belonging and appreciation for UiM trainees.

### Acknowledge the cognitive load of stereotype threat

UiM trainees also experience stereotype threat, described as being at risk of confirming, as self‐characteristic, a negative stereotype about one's group.^52^ This concept describes the fear held by members of a minoritized group that they may confirm negative stereotypes about their race through their own poor performance. For instance, if prior to performing a procedure a UiM trainee hears, “This may be hard for you.” This distress can provide undue mental and emotional stress on a resident, resulting in suboptimal academic and clinical performance. It is crucial to understand the external factors that create this cognitive dissonance, as Black, Latino, and Asian medical trainees were 10–20 times more likely to experience stereotype threat than non‐Hispanic White students.^53^ Structured discussions about stereotype threat at residency conference and DEI workshops can help create a supportive and inclusive environment where open communication about allyship for underrepresented groups is discussed.^53^ Additionally, a diverse faculty can foster a welcoming and stimulating environment for medical residents by providing relatable role models, promoting inclusive practices, and minimizing stereotype threat through the normalization of diverse perspectives and success stories.^53^

## MITIGATING DEPARTMENT‐LEVEL CHALLENGES

Intentional efforts at the department level should be made to foster an inclusive environment and address barriers to success for UiM trainees. These can be initiated by those in leadership roles such as vice chair of diversity, equity, and inclusion and carried out in partnership with the department chair as well as other institutional bodies (e.g., medical school, graduate medical education office) focused on advancing DEI.

### Recognize the “pull‐and‐push” dynamic

Expanding DEI initiatives to recruit UiM residents in conjunction with the unique challenges that UiM residents face creates a “pull‐and‐push” dynamic during their training (Table 1). While minoritized residents experience the pull into residency programs during recruitment with increased diversity efforts, this experience soon may be replaced by the pushing forces of isolation and exclusion by the factors discussed previously.^54^ Yet, despite feeling marginalized, UiM residents are pulled into serving on DEI committees and are taxed with being race and ethnicity ambassadors.^35^, ^55^ These roles may be important and rewarding for UiM trainees in academic medicine, but they occupy time and cognitive energy that potentially interfere with their ability to commit to other activities that are more traditionally recognized and rewarded by academic metrics such as research and teaching opportunities.^56^, ^57^ It must be a top priority for administrators at the GME and department levels to establish leadership positions for UiM trainees to engage in this work, that must come with protected time, opportunities to network with health system leaders, and appropriate titles and resources.^58^

**TABLE 1:** Push‐and‐pull effects on UiM trainees.

Pull effects (additional diversity‐related roles)	Push effects (challenges faced by UiM trainees)
Recruitment efforts UiM trainees often participate in recruitment activities to showcase program diversity	Racism Systemic racism in the training environment, creating barriers to inclusion and success
Appointment to DEI committees UiM trainees are frequently placed on DEI committees, adding extra responsibilities	Microaggressions/macroaggressions Daily, often subtle or overt, discriminatory remarks or actions that diminish trainees' sense of belonging
Featured on residency literature UiM trainees are often highlighted in residency promotional materials to demonstrate diversity	Unequal performance standards UiM trainees are sometimes held to higher or inconsistent standards compared to their peers
Assignment of DEI administrative tasks Administrative work for diversity initiatives is often disproportionately assigned to UiM trainees	Imposter syndrome UiM trainees may feel unworthy of their position due to implicit biases, questioning their competency
Involvement in DEI mentoring UiM trainees are frequently involved in mentoring roles related to DEI efforts, which can become an extra burden	Stereotype threat Fear of confirming negative stereotypes, leading to stress and reduced performance

Abbreviations: DEI, diversity, equity, and inclusion; UiM, underrepresented in medicine.

### Ensure equitable evaluations

Fair and equitable evaluations should be guaranteed for every resident, yet studies have shown that UiM residents are rated with lower competency scores than residents who do not identify as UiM.^59^, ^60^ Additionally, UiM trainees themselves perceive that they are held to a higher standard than non‐Hispanic White residents and that they are more likely to be punished or dismissed from residency than non‐Hispanic White residents when they struggle academically instead of being supported and mentored.^32^, ^61^ UiM women residents in both 3‐ and 4‐year EM residency programs were consistently rated lower in ACGME milestone assessments than White men residents.^62^ Residency program leadership should adopt measures to ensure equitable performance evaluations, which may include tracking data on resident evaluations by demographics, utilizing multisource feedback (e.g., from physicians, staff, and peers), and educating faculty on how to avoid biased language in feedback such as racially charged words or personality characteristics, as opposed to focusing on objective markers of performance.^63^ For example, instead of using a subjective interpretation, e.g., “The resident is unprofessional,” providing an objective example, e.g., “The resident was late to shift by 30 minutes” (Table 2).

**TABLE 2:** Recommendations for supporting UiM trainees.

Individual‐level recommendations	
1. Recognize and mitigate the impact of homophily	Promote inclusion in social events and professional opportunities. Foster a sense of belonging through mentorship and community‐building activities.
2. Know the cumulative harm of microaggressions	Implement training to reduce microaggressions as part of the residency curriculum.
3. Understand the impact of racism	Enforce zero‐tolerance policies for racism. Provide reporting system for residents affected by racist encounters.
4. Recognize the external cues that feed imposter syndrome	Build an inclusive environment that instills a sense of belonging and appreciation for UiM trainees.
5. Acknowledge the cognitive load of stereotype threat	Create an inclusive environment where UiM residents feel valued and supported.
Departmental‐level recommendations	
1. Recognize the pull‐and‐push dynamic	Ensure ongoing support and inclusion for UiM residents beyond recruitment. Establish consistent support structures throughout residency.
2. Ensure equitable evaluations	Implement multisource feedback systems and track evaluations by demographics. Train evaluators to recognize and avoid biased language and assessments.
3. Recruit UiM faculty	Develop competitive recruitment packages for UiM faculty. Provide protected time and resources for mentorship and DEI work to prevent the minority tax.
4. Train all faculty to mentor across differences	Implement training for all faculty on cross‐cultural mentorship and how to support UiM residents effectively.
Institutional‐level recommendations	
1. Establish health system community partnerships	Form longitudinal partnerships with the communities served. Involve community members in advisory boards for public health projects and community‐based participatory research.
2. Ensure adequate resources for increasing accessibility	Invest in multilingual interpreter services and other resources to serve diverse populations effectively.
3. Evaluate the training environment strategically	Hold training programs accountable for inclusive practices that prioritize the recruitment, retention, and support of diverse residents such as monitoring the number of UiM residents at an institution, supporting GME‐wide DEI events, and examining residency remediation proceedings for potential bias.
4. Establish formal faculty development on health equity	Lead faculty training sessions at the GME level to equip faculty with the knowledge, skills, and agency to address racist practices.
5. Incorporate wellness measures	Consider innovative solutions for resident wellness such as virtual programming, protected time for scheduling primary care and mental health appointments, and assigning mental health and primary care providers to trainees before they begin residency training.

Abbreviations: DEI, diversity, equity, and inclusion; GME, graduate medical education; UiM, underrepresented in medicine.

### Recruit UiM faculty

Mentorship is important for all trainees, but is particularly crucial for UiM residents as they must navigate the effects of institutional, interpersonal, and internalized racism while completing already rigorous training programs.^54^, ^64^ Institutions must prioritize recruiting faculty from diverse backgrounds to not only improve patient care but to also adequately support UiM learners throughout residency training.^65^ UiM faculty provide valuable mentoring opportunities and help to create a sense of community for UiM trainees.^66^ However, UiM trainees have expressed difficulty finding racially concordant mentors.^67^ Health systems must provide competitive recruitment packages for UiM faculty that include protected time and resources for academic pursuits, mentorships opportunities, and career advancement. Appropriate compensation for DEI work and/or titles can mitigate the “minority tax” for these faculty, which often serves as a barrier to academic advancement, productivity, and wellness.^68^, ^69^, ^70^, ^71^, ^72^ Collaboration between hospital and GME leaders is instrumental in garnering financial support for these initiatives and investing in a diverse physician workforce. Faculty recruitment committees should develop competitive recruitment packages for UiM faculty that provide protected time and resources for mentorship and DEI work to prevent the minority tax.

### Train all faculty to mentor across differences

It is critical that all faculty be trained to mentor UiM trainees, despite a potential lack of racial and gender congruity. This is not only due to the paucity of available UiM faculty members who can serve as potential mentors but also due to the importance of providing residents with mentors who may have expertise in similar areas of interest or who exemplify a desired career trajectory. To effectively work with UiM trainees, mentors would benefit from mentor training models that provide guidance detailing how to create a mentoring relationship that not only invites trust and openness but also acknowledges the negative impact of racism, microaggressions, and other factors discussed above on medical training for UiM trainees.^73^, ^74^, ^75^ Faculty trainings organized at the department level for professional development should include topics such as evidence‐based education on ally identity development and interactive upstander training to equip faculty with the skills to support UiM trainees.^76^, ^77^ Faculty training on cross‐cultural mentoring should be provided to assure UiM mentees have access to multiple mentors who have shared interests.

## NAVIGATING INSTITUTION‐LEVEL CHALLENGES

Interventions to support UiM trainees cannot be limited to the department level. Academic institutions, hospitals, health systems, medical schools, and senior leaders should support and engage in DEI initiatives to promote equity in both medical education and patient care. These efforts can be synergistic with departmental efforts to create an environment where UiM trainees thrive. Given the body of evidence supporting that a diverse physician workforce improves the experiences and outcomes of racially and ethnically minoritized patients, health systems have clear clinical, financial, and moral incentives to recruit and retain UiM physicians.^4^, ^5^, ^6^, ^9^ Initiatives involving leaders of both the health system and GME can ultimately function to support and empower UiM residents during their training.

### Establish health system community partnerships

For UiM residents to thrive, health systems must invest in the communities that they serve, forming longitudinal partnerships that provide trainees the opportunity to meaningfully engage with patients and their families both clinically and in the community. These initiatives, while potentially initiated by trainees and faculty members, should be supported by emergency department leaders such as the vice chair of diversity, equity, and inclusion and institutional leaders that may have roles in community engagement or DEI. Before residents are recruited to these institutions, training programs can incorporate community members in the interview process and admission decisions so their voices are prioritized in the selection process for doctors who will serve them.^78^, ^79^ Appropriately compensated advisory boards involving community members can influence care delivery. When given opportunities to share experiences in influential forums, these advisory boards can help to keep health systems accountable and center community needs as it pertains to patients’ access to and receipt of care. Health systems should financially invest in the surrounding community by employing members of the community and supporting minority‐owned businesses, which build wealth among historically oppressed groups and additionally decrease barriers for UiM students to enter medicine.^80^, ^81^ Academic institutions should form longitudinal partnerships with the surrounding communities to involve community members in advisory boards for public health projects and community‐based participatory research.

### Ensure adequate patient resources for increasing accessibility

UiM trainees who are invested in serving minoritized patient populations value institutions that have the resources to serve them.^80^, ^82^ For example, health systems should invest in building accessible, inclusive environments for patients with limited English proficiency, low health literacy, and other challenges with interventions such as multilingual signage, written materials and robust interpreter services.^83^, ^84^ This may also include financial literacy, financial support, and social work resources. Collaboration should take place at the department, GME, and hospital levels and center on how a diverse and fulfilled workforce will ultimately better the institution and improve patient care. These collaborative efforts between trainees, faculty members, departmental and institutional leaders, and community members can help with advocating for resource allocation for these efforts.

### Evaluate the training environment strategically

The GME office oversees all ACGME‐accredited residency training programs at an institution, with the designated institutional officer (DIO) as the leader. Leveraging the GME office as it pertains to DEI efforts provides the opportunity to create consistency and motivation to lead DEI initiatives and create fellowship among UiM trainees across training programs. Commitments to DEI by departments and institutions must go beyond mission statements and be accompanied with concrete, measurable, actionable, and timely goals with strategic planning. This requires data collection and DEI‐specific program evaluation, which should be required by the institution's GME office to hold training programs accountable for inclusive practices and to prioritize the recruitment, retention, and support of diverse residents.^85^, ^86^ Possible measures include monitoring the number of UiM residents at an institution, supporting GME‐wide DEI events, and examining residency remediation proceedings for potential bias. Using measurable goals and identifying how they align with health system priorities, GME leaders (i.e., DIOs) can collaborate with hospital and departmental leaders to obtain funding for staff support to gather these data; protected time for physicians to engage in fellowship, mentorship, or community outreach; and curricular support for incorporating DEI content and inviting speakers of diverse backgrounds across all training programs. Affinity groups and cross‐specialty mentorship programs can serve as additional resources for UiM residents, providing opportunities to build community, access support, and foster connections that beyond their immediate department.^86^ Giving UiM trainees a space to share their perspectives, listening to their experiences, and educating oneself on historical context and antiracist practices is essential.^87^

Lastly, it is the responsibility of the GME office to investigate bias in remediation and evaluate disparities in resident attrition by training program in order to pinpoint areas where inequities may manifest and be addressed.^88^ At this level, there should be an official pathway where discrimination and racism can be reported, confronted, and given the necessary attention such that both individual instances and structures are addressed appropriately.

### Establish formal faculty development on health equity

Supporting UiM trainees requires that the faculty members involved in their education and training are well‐versed in topics of health equity.^66^ Given that many faculty were trained in systems that were dominated by White and other historically majority perspectives, they must revisit the truths about racism in medicine both past and present that are often omitted from the curricula.^89^ Faculty development must include topics such as critical race theory and upstander training for micro‐ and macroaggressions. These training sessions should be led at the GME level to equip faculty with the knowledge, skills, and agency to address racist practices.^90,^, ^91^ To do this and advise on other matters surrounding DEI, a position in the GME office such as a UiM program manager should be held by one with DEI expertise, ideally with ties to the community in which the institution resides.

### Incorporate wellness measures

Finally, frameworks must be in place to support the well‐being of UiM residents that go beyond cursory wellness events, such as complimentary food or yoga and meditation. Microaggressions during medical training have been associated with positive screenings for depression and lower rates of academic satisfaction.^92^ Yet despite a decline in health and wellness during residency, many trainees do not seek medical care due to a demanding work schedule that prevents access to a primary care provider during regular clinic hours or due to concerns about their own privacy.^93^ Innovative solutions may include virtual programming options (e.g., residency conference, alternatives to in‐person meetings) and protected time for scheduling primary care and mental health appointments. Assigning mental health and primary care providers to trainees before they begin residency training has been implemented as a supportive strategy.^94^

## PITFALLS AND MITIGATION STRATEGIES

Interventions to dismantle traditionally majority‐serving processes and rebuild an infrastructure that supports UiM trainees is necessary but challenging.^25^ It requires moving past superficial solutions that fail to provide meaningful change, questioning the status quo and existing policies and critically reflecting on whether interventions are addressing the needs of UiM trainees. Three themes emerge as common pitfalls when pursuing this work: lack of vision, lack of resources, and performative allyship.

### Lack of vision

Lack of vision in equity work can manifest as shortsighted interventions that do not truly address underlying causes of inequity and discrimination against UiM trainees. Establishing a meaningful vision requires a strong mission statement that is reflective of the desire to address root causes of inequity, focuses on concrete and aspirational statements rather than declarative messaging (e.g., “We don't discriminate …”), acknowledges current efforts being taken to promote DEI, and is followed by actionable steps including a budget and funding sources to achieve the established goals.^86^, ^95^ Mission statements that explicitly emphasize increasing diversity can provide clarity on a residency program's core values, serving as a foundation to integrate these principles into the program's culture and curriculum. When a well‐defined DEI‐focused mission is embedded in a program's daily operations, it can lead to measurable positive outcomes. For instance, medical schools with mission statements targeting increased representation of underrepresented minority groups have demonstrated higher graduation rates among these populations.^96^ However, mission statements without sufficient funding, administrative backing, and genuine implementation risk becoming merely performative.

### Lack of resources

Executing an impactful vision requires institutional investment of time, resources, and funds. Financial compensation, protected time, and pathways to promotion for DEI work for faculty and trainees are integral to minimize the implementation of minority tax.^97^ Voluntary DEI work without compensation furthers inequitable treatment, taking away from activities that contribute to academic productivity, promotion, and wellness. Institutions should hire those with experience and expertise, as well as administrative support staff, to alleviate this burden and more efficiently accomplish these tasks.^55^, ^56^

### Performative allyship

Performative allyship occurs when individuals or organizations publicly profess solidarity with a marginalized group but then take actions that offer no benefit or can be harmful for that group.^98^, ^99^ In practice, performative allyship appears as (1) superficial engagement (e.g., social media posts without commitment to longitudinal advocacy work), (2) remaining quiet or not actively addressing instances in which problematic sentiments are expressed or inequitable policies are enacted, and (3) expecting recognition or profiting from their allyship work. In academic medicine, performative allyship can be noted in programs that make public proclamations about their commitment to diversity and inclusion yet fail to be self‐critical or make earnest attempts to evaluate what changes may be necessary to assure that the training environment is equitable and safe for all trainees.^100^

Another example of performative allyship includes placing UiM people in leadership positions without funding or mentorship to set them up for success. During the hiring process, department leadership should avoid a professional form of performative allyship that can occur with the phenomenon of the “glass cliff” for UiM candidates. The glass cliff describes the tendency for women and underrepresented candidates to be appointed to leadership positions during times of crisis, with their recruitment into these positions being a mark of success for the program.^101^ However, the appointment of a faculty member to a DEI role is not enough. Without the necessary commitment to further departmental change after the appointment of this UiM leader, there is a risk that this appointee may be set up for failure, as major change requires longitudinal commitment at all levels. Taking on a DEI position as an interdepartmental promotion or as a new hire without widespread support could place the faculty member as a target for blame if major changes are not achieved in a short time period, thus making them “fall off the glass cliff.” Much like the commitment to retention that needs to exist when recruiting UiM residents, there should also be a similar commitment to assuring that UiM faculty are adequately supported so they may have the time and resources to take part in supporting underrepresented trainees at their institution.^102^, ^103^, ^104^, ^105^, ^106^, ^107^, ^108^

Successful allyship at the departmental level hinges on authentic and genuine commitment to cultivate an environment of support and belonging. In the most powerful form of allyship, departmental leaders use their own privilege and power to advocate for marginalized groups. It is important to note that allyship is not self‐defined—one's work and efforts must be recognized by the individuals or communities one seeks to support. It is also an active, ongoing process of building relationships based on trust, consistency, and accountability.^97^ Evidence‐based strategies for authentic allyship include (1) self‐examination and critical analysis of individual and institutional beliefs, policies, and practices; (2) providing spaces, forums, and community for underrepresented groups and individuals; (3) identifying and calling out racism and discrimination; (4) establishing mentorship; and (5) using privilege to advocate and enact change.^100^

## CONCLUSIONS

Efforts to create a diverse emergency medicine workforce must go beyond underrepresented in medicine resident recruitment during the application season. Residency programs must place equal importance on creating a supportive and safe training environment for underrepresented in medicine trainees throughout their residency experience. Strategies for creating an environment where residents can flourish are multitiered, requiring efforts at the individual, departmental, and institutional levels. These efforts must aim to challenge traditional majority‐serving structures and be data‐driven, supported by leadership, and responsive to resident and faculty feedback to create a culture that is equitable and inclusive.

Our recommendations are specifically directed toward residency program, department, and institutional leaders, as they are positioned to implement impactful and sustainable changes within the residency training environment. Future work will focus on addressing biases involving the broader emergency department staff, including patients, nurses, and advanced practice providers.

## AUTHOR CONTRIBUTIONS

Study concept and design: All authors. Drafting of the manuscript: All authors. Critical revision of the manuscript for important intellectual content: Rosemarie Diaz and Jessica Faiz.

## CONFLICT OF INTEREST STATEMENT

The authors declare no conflicts of interest.
